# Molecular signatures of anthroponotic cutaneous leishmaniasis in the lesions of patients infected with *Leishmania tropica*

**DOI:** 10.1038/s41598-020-72671-7

**Published:** 2020-10-01

**Authors:** Nasrin Masoudzadeh, Malin Östensson, Josefine Persson, Vahid Mashayekhi Goyonlo, Christopher Agbajogu, Yasaman Taslimi, Reza Erfanian Salim, Farnaz Zahedifard, Amir Mizbani, Housein Malekafzali Ardekani, Bronwyn M. Gunn, Sima Rafati, Ali M. Harandi

**Affiliations:** 1 Department of Immunotherapy and Leishmania Vaccine Research, Pasteur Institute of Iran, Tehran, Iran; 2 Department of Microbiology and Immunology, Institute of Biomedicine, Sahlgrenska Academy, University of Gothenburg, Gothenburg, Sweden; 3 Cutaneous Leishmaniasis Research Center, Mashhad University of Medical Sciences, Mashhad, Iran; 4 Noor Eye Hospital, Tehran, Iran; 5 ETH Zurich, Zurich, Switzerland; 6 Ragon Institute of MGH, MIT, and Harvard University, Cambridge, MA 02139 USA; 7 Vaccine Evaluation Center, BC Children’s Hospital Research Institute, The University of British Columbia, Vancouver, Canada; 8 Present Address: Paul G. Allen School of Global Animal Health, Washington State University, Pullman, WA 99164 USA

**Keywords:** Immunology, Molecular biology, Systems biology

## Abstract

Anthroponotic cutaneous leishmaniasis (CL) caused by *Leishmania tropica* (*L. tropica*) represents a public health challenge in several resource poor settings. We herein employed a systems analysis approach to study molecular signatures of CL caused by *L. tropica* in the skin lesions of ulcerative CL (UCL) and non-ulcerative CL (NUCL) patients. Results from RNA-seq analysis determined shared and unique functional transcriptional pathways in the lesions of the UCL and NUCL patients. Several transcriptional pathways involved in inflammatory response were positively enriched in the CL lesions. A multiplexed inflammatory protein analysis showed differential profiles of inflammatory cytokines and chemokines in the UCL and NUCL lesions. Transcriptional pathways for Fcγ receptor dependent phagocytosis were among shared enriched pathways. Using *L. tropica* specific antibody (Ab)-mediated phagocytosis assays, we could substantiate Ab-dependent cellular phagocytosis (ADCP) and Ab-dependent neutrophil phagocytosis (ADNP) activities in the lesions of the UCL and NUCL patients, which correlated with *L. tropica* specific IgG Abs. Interestingly, a negative correlation was observed between parasite load and *L. tropica* specific IgG/ADCP/ADNP in the skin lesions of CL patients. These results enhance our understanding of human skin response to CL caused by *L. tropica*.

## Introduction

Leishmaniasis is a complex neglected tropical infectious disease that is endemic in 98 countries with about 0.7 to 1.3 million estimated cases every year. Over 20 different species of protozoa of the *Leishmania* genus cause three main clinical forms of the disease (cutaneous, mucocutaneous and visceral), of which cutaneous leishmaniasis (CL) is the most common form^1–3^. Globally, around 75% of all CL cases are reported in ten countries: Afghanistan, Syria, Sudan, Ethiopia, Algeria, Brazil, Colombia, Costa Rica, Peru and Iran^4^. Anthroponotic CL (ACL), caused by *L. tropica,* and zoonotic CL (ZCL), caused by *L. major*, are two pre-dominant variants of CL that are highly prevalent in the endemic countries and represent a major health problem in the Old World^5,6^.

ACL is mostly spread in urban areas^7,8^ through transmission from the infected female sand fly vector, *Phlebotomus sergenti*, to humans^5^. Cutaneous lesions caused by *L. tropica* are characterized by papules and nodules (non-ulcerative CL; NUCL), which in some cases may develop progressively to an ulcerative form (ulcerative CL; UCL) with ulcerated, volcanic, epidermis^9,10^. The closely related *Leishmania* genus *L. aethiopica*^11,12^ manifests comparable dermatopathology in the forms of localized CL with ulcerated epidermis, and diffuse CL with intact epidermis^13,14^.

The CL lesions caused by *L. tropica* often have a prolonged healing time (6–15 months) and tend to be more resistant to treatment^15^ compared to CL caused by *L. major*. Only a limited number of human studies have focused on understanding immune mechanisms involved in *L. tropica*-induced CL^9,16,17^.

Recently, advanced whole genome transcriptomics technology combined with systems biology approaches have been used to identify transcriptional changes in human tissues in response to pathogens^3,18–20^. Two recent studies have reported host transcriptional responses in the skin lesions of patients infected with *L. braziliensis*^19,21^. We have recently, for the first time, reported the gene expression profile in the skin lesions of a limited number of patients infected with *L. tropica*^22^, and demonstrated that a number of immune-related genes, including inflammatory and immunoglobulin genes, were up-regulated within lesions.

**Figure 1:**
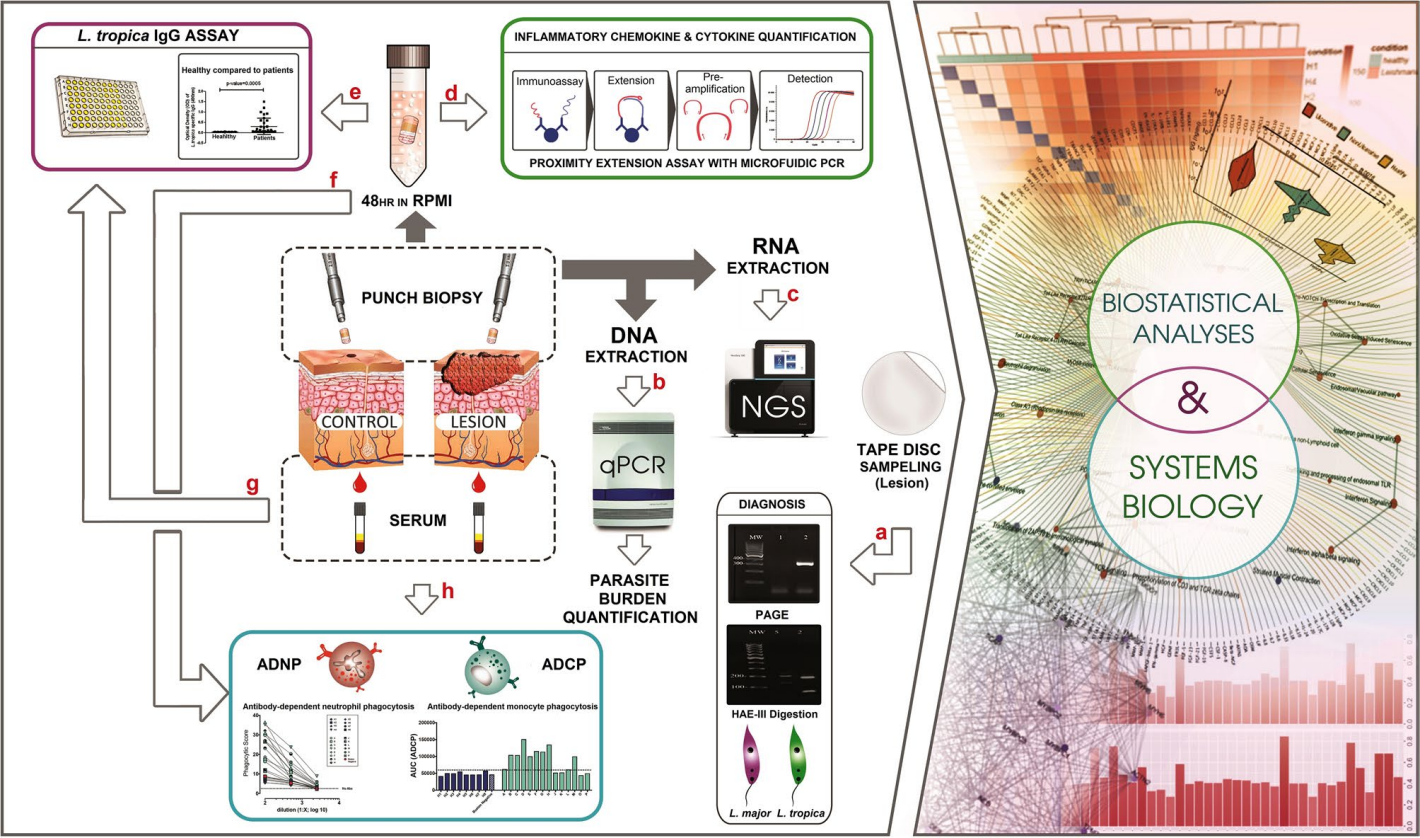
Schematic representation of the study work flow and methodology. Assays used in the study included. (a) The ITS1 PCR–RFLP assay for diagnosis of *L. tropica* patients. (b) Quantitative polymerase chain reaction **(**qPCR) analysis for quantification of *L. tropica*. (c) NextSeq 500 platform based RNA-seq method for investigation of whole genome transcription in biopsy samples. (d) Proximity extension assay (PEA) in biopsy supernatants for inflammatory protein assessment. (e, g) Enzyme-linked immunosorbent assay (ELISA) in biopsy supernatants and serum samples for *L. tropica* specific IgG antibody assessment. (f, h) Antibody-dependent neutrophil phagocytosis (ADNP) and Antibody-dependent cellular phagocytosis by human monocytes (ADCP) in biopsy supernatants and serum samples.

Herein, we employed RNA sequencing analysis, high throughput inflammatory protein assay, antibody-dependent phagocytosis assays combined with a systems biology approach to pinpoint molecular signatures of CL lesions in *L. tropica* infected patients with ulcerative (UCL) and non-ulcerative (NUCL) manifestations (Fig. 1)*.* Results revealed functional transcriptional pathways shared or otherwise exclusive to the skin lesions of the UCL and NUCL patients. In line with the transcriptomics results, the presence of inflammatory proteins, and *L. tropica* specific FcγR dependent phagocytosis activity in the skin lesions of the CL patients were documented. These results enhance our understanding of host response to anthroponotic CL caused by *L. tropica* in human skin, and may inform rational design of novel intervention approaches to counter CL in humans.

## Results

### Diagnosis and parasite load in skin lesions of CL patients

Using a non-invasive sampling method combined with *L. tropica* specific PCR^23^, all CL patients were diagnosed as *L. tropica* positive (Fig. S1). The *L. tropica* patients were dermatologically classified based on the clinical appearance of their lesions into UCL with ulcerated epidermis (n = 8) and NUCL with intact epidermis (n = 9). Clinical characteristics of the UCL and NUCL patients, including sex, age, lesion size, illness duration and number of lesions, are presented in Table S1. Although UCL and NUCL represent dermatologically differentiated manifestations, we did not find any significant difference between UCL and NUCL as pertain to the lesion size, illness duration or the number of lesions (data not shown).

*L. tropica* burden in the skin lesions was determined by a *L. tropica* kDNA minicircles specific qPCR^24^. The parasite loads in the lesions of NUCL patients was found to be approximately four times higher than those of the UCL group (NUCL 629.8 ± 393.53, UCL 149.17 ± 106.83), albeit the difference was not statistically significant (*p* = 0.29).

### RNA sequencing analysis of the lesions in UCL and NUCL patients infected with *L. tropica* and healthy skin biopsies

**Figure 2:**
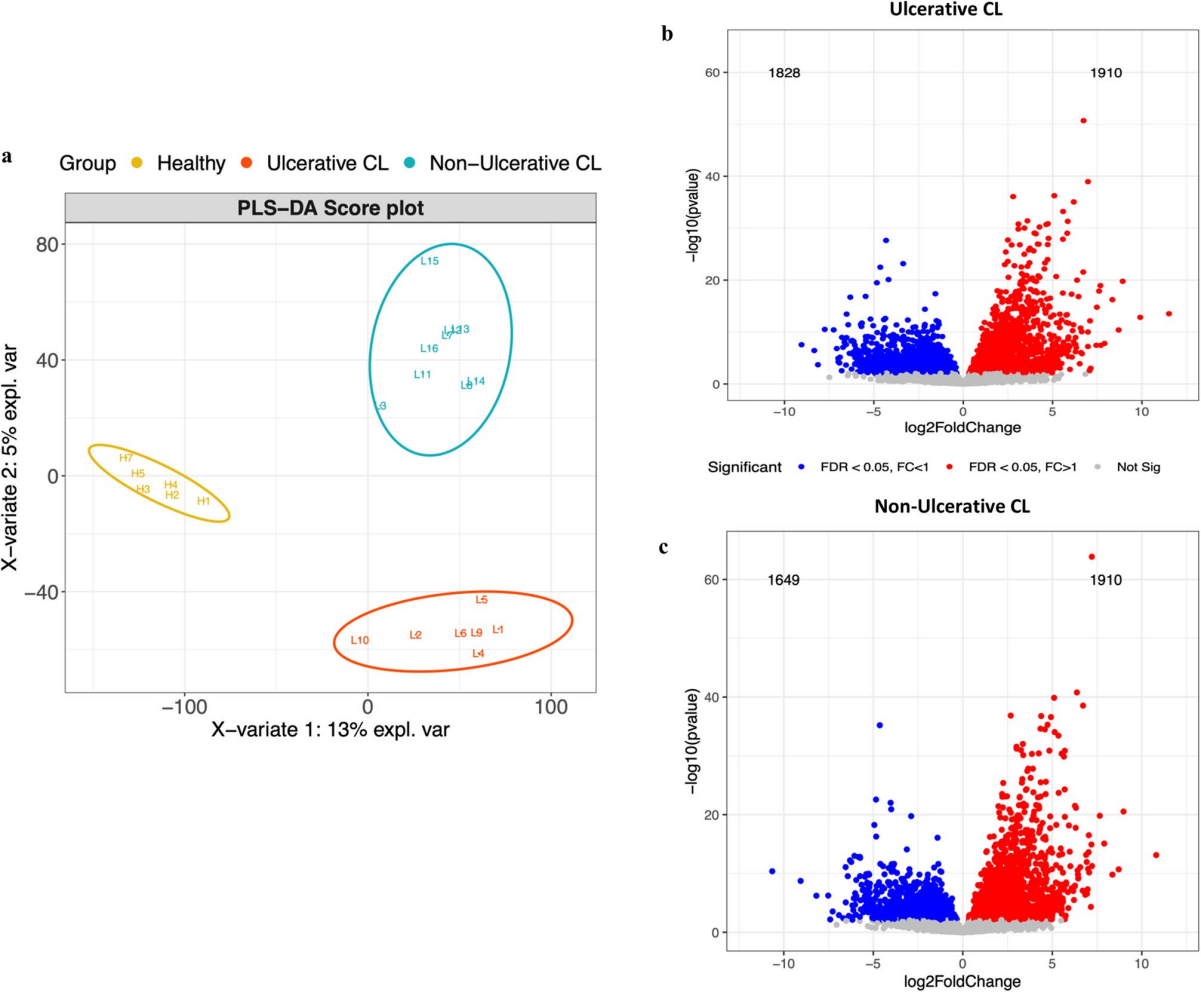
Transcriptomics analysis of skin lesions in ulcerative cutaneous leishmaniasis (UCL) and non UCL (NUCL) of *L*. *tropica-*infected patients, and healthy skins. (**a**) Partial least squares-discriminant analysis (PLS-DA) modeling was applied for classification of UCL and NUCL lesions and skin biopsies from healthy individuals. Volcano plots of UCL (**b**) and NUCL (**c**) based on DESeq2 results. Dots in red (Log FC > 1) and blue (Log FC < 1) represent differentially expressed genes with adjusted *p*-value < 0.05. All three figures were created using R version 3.5.1, PLS-DA with R package mixOmics 6.6.2 and volcano plots using R package ggplot2 3.2.1.

RNA extracted from skin lesion biopsies of 16 *L*. *tropica*-infected CL patients, including seven UCL and nine NUCL, and six skin biopsies of healthy individuals were subjected to whole genome sequencing. Partial least squares-discriminant analysis (PLS-DA) model was used to define the pattern of gene expression among the UCL, NUCL and healthy skin samples. The analysis showed a clear separation among the three groups (Fig. 2a). The differentially expressed genes (DEGs), based on DESeq2 analysis, were identified in the lesions from the UCL and the NUCL patients relative to healthy skins (Fig. 2b,c). A total of 3738 and 3559 genes were differentially expressed (using an adjusted *p*-value cut-off of 0.05) in the lesions of the UCL and NUCL compared to healthy skins, respectively. Of these genes, 2663 DEGs were common between the UCL and NUCL groups. The expression of 1075 and 896 DEGs was uniquely observed in the lesions of the UCL and the NUCL patients relative to healthy samples, respectively.

### GSEA identifies unique gene pathways in the lesions of UCL and NUCL patients

A gene set enrichment analysis (GSEA) was performed on the gene expression data from the UCL and NUCL lesions compared with the healthy skins to pinpoint biological functions related to groups of genes by a Reactome database. Results from GSEA along with statistical parameters, including ES, NES and adjusted *p*-values are presented in Table S2.

**Figure 3:**
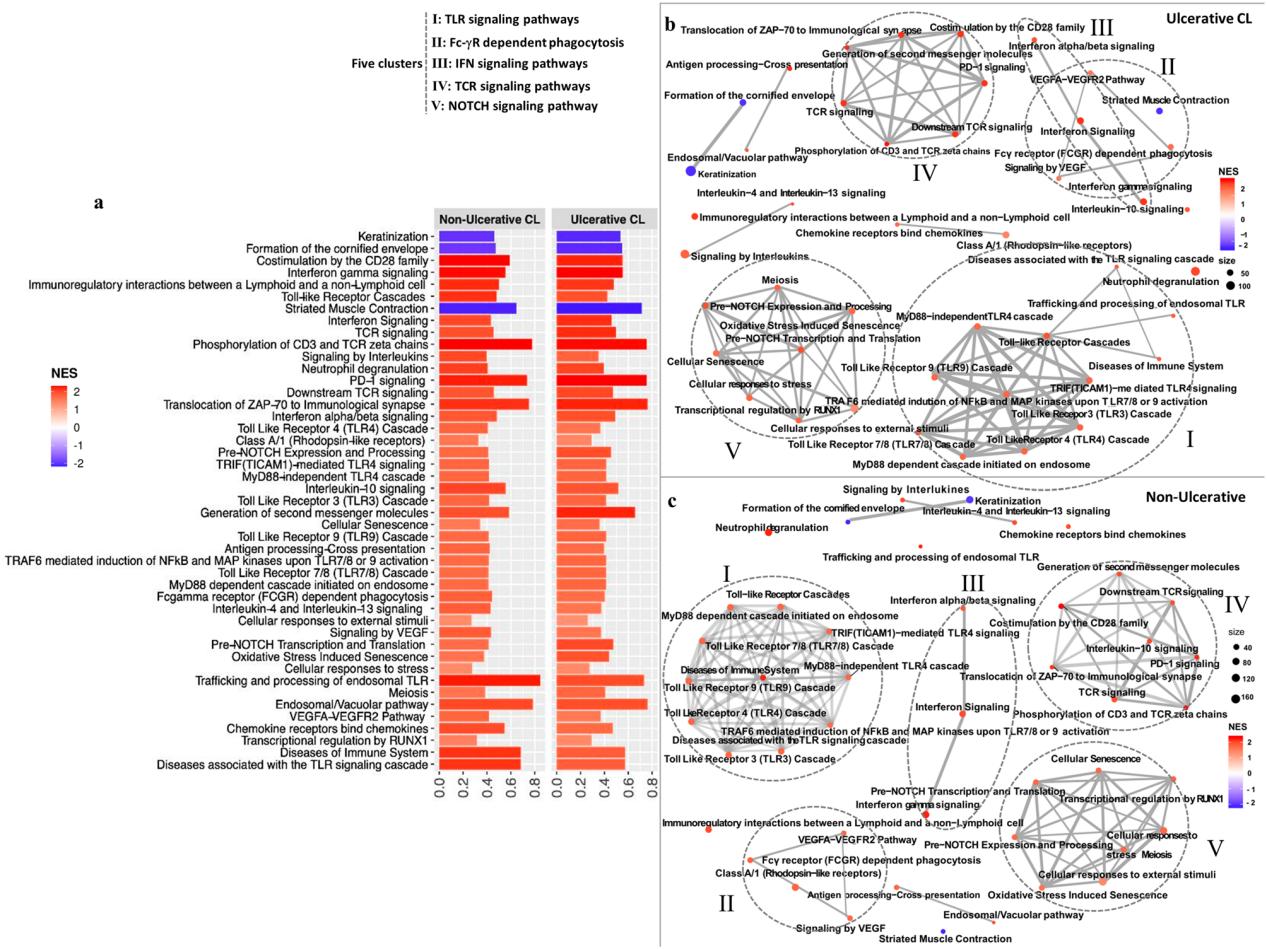
Common enriched GSEA pathways in the lesions of UCL and NUCL patients. Bar plot (**a**) and enrichment map plot (**b**,**c**) of GSEA revealed enrichment of 45 common pathways in the skin lesions of *L. tropica*-infected UCL and NUCL patients. In (**b**) and (**c**), dash circles denote five main clusters of enriched pathways. The color and length of the boxes represent normalized enrichment scores (NES), and the number of genes mapped to the indicated pathways, respectively. Positive and negative NES show up- and down-regulated pathways in the skin lesions of the CL patients compared with skin biopsies of healthy individuals, respectively**.** For the pathway analysis ReactomePA R package version 1.28.0 was used^69^. This package version uses Reactome version 70 (https://reactome.org/about/news/142-version-70-released). All figures were created using R version 3.5.1.

The analysis identified 100 and 49 enriched Reactome pathways (adjusted *p*-values < 0.05) in the skin lesions of UCL and the NUCL groups, respectively, when compared with healthy skin samples. Of these, 55 enriched pathways were unique for the UCL lesions (Fig. S2a), while only four enriched pathways were uniquely enriched with positive score in the NUCL lesions (Fig. S2c). Forty-five biological pathways were common between the skin lesions of the UCL and NUCL patients (Fig. 3a).

Several of the highly enriched pathways in the skin lesions of UCL patients were related to gene expression, epigenetic regulation of gene expression and signal transduction (Fig. S2a,b). The “gene expression pathway” includes several genes “RNA Polymerase I promoter clearance”/promoter opening/transcription”, “RUNX1 regulates genes involved in megakaryocyte differentiation and platelet function”, “RUNX1 regulates transcription of genes involved in differentiation of hematopoietic stem cells”, “Gene silencing by RNA”, and “Transcriptional regulation by small RNAs”. These pathways could potentially contribute to parasite survival by host immune silencing mechanisms, non-coding RNAs network manipulation and transcriptional arrest of the protein coding genes in macrophages^25–27^. Further studies are however needed to pinpoint the function of these pathways in pathogenesis of, and immunity to, leishmaniasis. Pathways linked to epigenetic regulation that were positively enriched in the UCL data consists of “DNA methylation” and “PRC2 methylates histones and DNA”. “Positive epigenetic regulation of rRNA expression” and “SIRT1 negatively regulates rRNA expression”, were also positively enriched in the UCL group.

Furthermore, positive enrichment of several pathways involving signal transduction was observed in the UCL group. This includes nuclear receptors (“ESR-mediated signaling”), WNT (“Formation of the β-catenin: TCF trans activating complex”), G protein-coupled receptors (GPCR) (“Gα (s) signaling events”)-mediated signaling as well as RHO GTPase effectors (“RHO GTPases activate protein kinases N (PKNs)”, “Activated PKN1 stimulates transcription of androgen receptor regulated genes kallikrein 2 (KLK2) and KLK3”).

Pathways involved in B-cell development and activation, including “Signaling by the B Cell Receptor (BCR)”, “Downstream signaling events of BCR” and “Activation of NF-κB in B cells”, were also positively enriched in the UCL group, consistent with our prior analysis of DEGs in skin lesions^22^.

In the NUCL lesions, pathways related to nuclear structure and mitotic anaphase such as “Initiation of Nuclear Envelope Reformation”, “Nuclear Envelope Reassembly”, “Transcriptional Regulation by TP53”, and the phagocytosis -related pathway “Regulation of actin dynamics for phagocytic cup formation” were uniquely enriched with positive score (Fig. S2c,d).

### GSEA revealed common pathways up-regulated in UCL and NUCL lesions

To identify functional clusters of genes and biological pathways that are over-represented in the sequencing data of the skin lesions of UCL and NUCL patients, gene set enrichment analysis (GSEA) was performed as explained in “Methods” section. The enriched GSEA pathways were classified into five main clusters (Fig. 3a–c). Among these, several pathways involved in innate and inflammatory responses were noted. This includes Toll-like receptor (TLR) signaling, with “TIR-domain-containing adapter molecule (TICAM1)-mediated TLR4 signaling”, “TLR3 cascade”, “TLR4 cascade”, “TLR7/8 cascade”, “TLR9 cascade”, “MyD88-independent TLR4 cascade”, “MyD88 dependent cascade initiated on endosome”, “Trafficking and processing of endosomal TLR”, and “Diseases associated with the TLR signaling cascade”.

Two additional small pathway clusters were identified, which among others include “Fc-γ receptor (FcγR) dependent phagocytosis”, “Signaling by vascular endothelial growth factors (VEGF)”, “VEGFA-VEGFR2 pathway” as well as IFN signaling pathways “IFN-α/β signaling” and “IFN-γ signaling”.

“Phosphorylation of CD3 and TCR ξ chains”, “Translocation of ZAP-70 to immunological synapse”, “Generation of second messenger molecules”, “Downstream TCR signaling pathways”, “Co-stimulation by the CD28 family” and the negative regulator of T cells “PD-1 signaling”, all associated with the T-cell receptor (TCR) complex, were also among commonly positive enrichment pathways in both groups. Pathways for NOTCH signaling (“Pre-NOTCH expression and processing”, “pre-NOTCH transcription and translation”), and those involved in “Cellular responses to stress”, “Cellular senescence and “Oxidative stress induced senescence” were positively enriched in the UCL and NUCL groups.

In addition, pathways involved in "Signaling by interleukin (IL)s", "IL-4 and IL-13 signaling", "Antigen processing-cross presentation", "Endosomal/Vacuolar pathway", "Chemokine receptors bind chemokines", "Class A/1 (Rhodopsin-like receptors)", "Neutrophil degranulation", "IL-10 signaling" and "Immunoregulatory interactions between a lymphoid and a non-lymphoid cell" were positively enriched in both the UCL and the NUCL groups (Fig. 3b,c).

GSEA analysis also identified three common negatively enriched pathways associated with skin pathophysiology, namely “Keratinization”, “Formation of the cornified envelope” and “Striated muscle contraction” (Fig. S2e–j).

### Protein expression pattern of inflammatory cytokines/chemokines in the skin lesions

**Figure 4:**
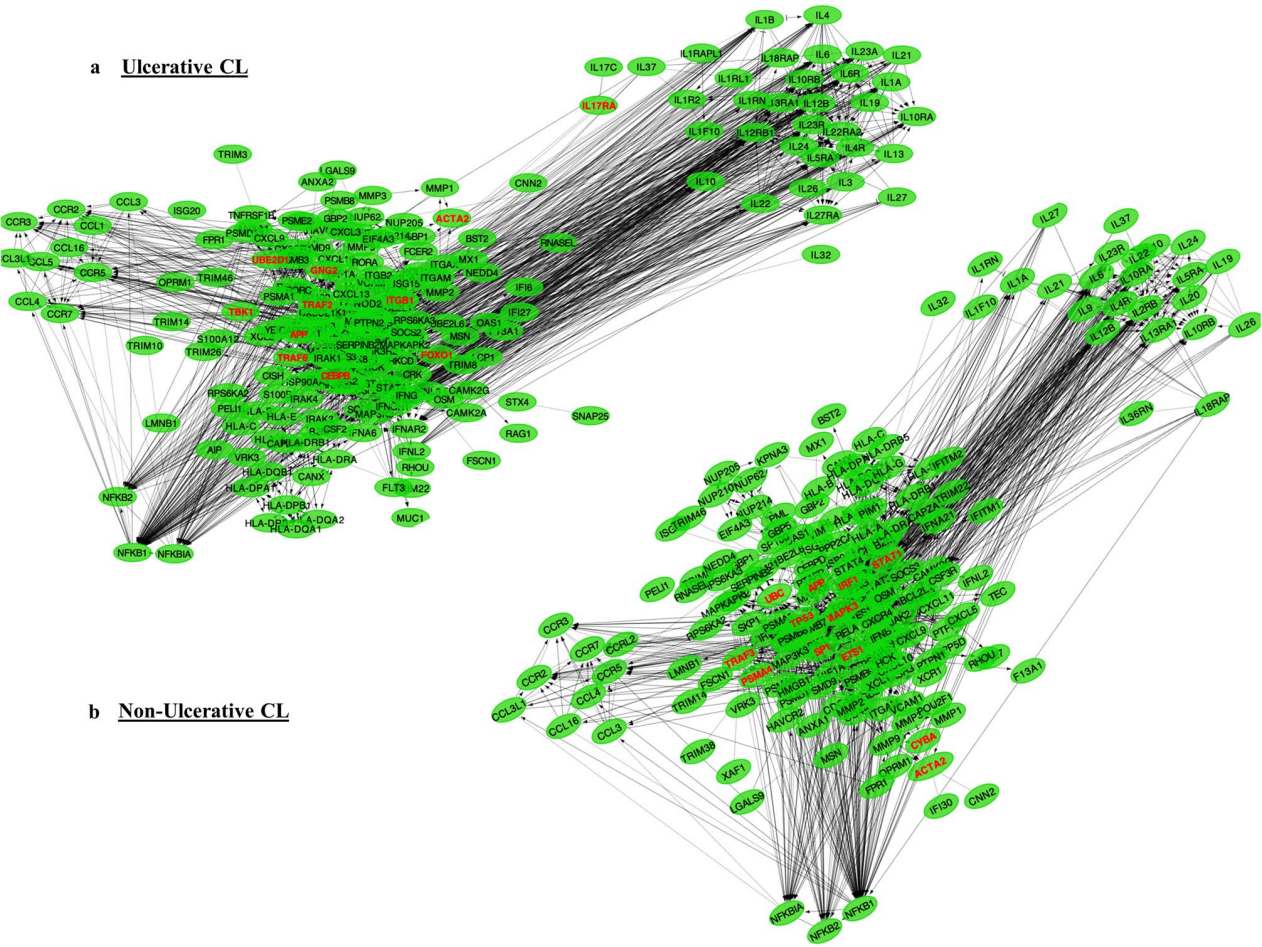
Gene networks combined by the common GSEA pathways. The GSEA results revealed significant transcriptional changes of several pathways involved in inflammatory responses in the *L. tropica* infected lesions, including “Chemokine receptors bind chemokines” , “Signaling by Interleukins”, “IL-10 signaling”, “IL-4 and IL-13 signaling” “IFN-γ signaling”, “IFN-α/β signaling”, and “IFN Signaling”, for UCL (**a**) and NUCL (**b**). This figure was created using Cytoscape version 3.7.2 and the Reactome FI plugin version 7.2.0.

**Figure 5:**
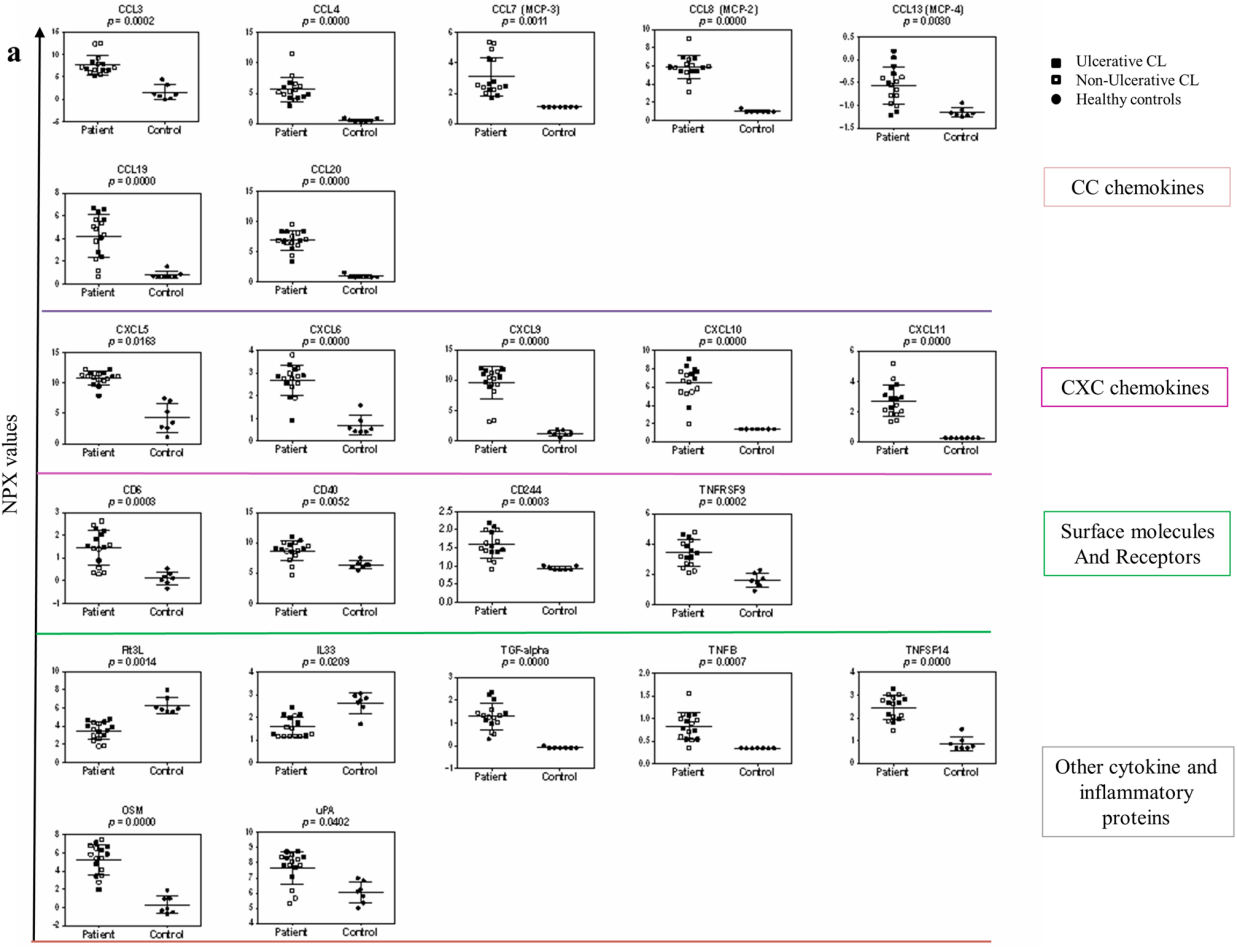

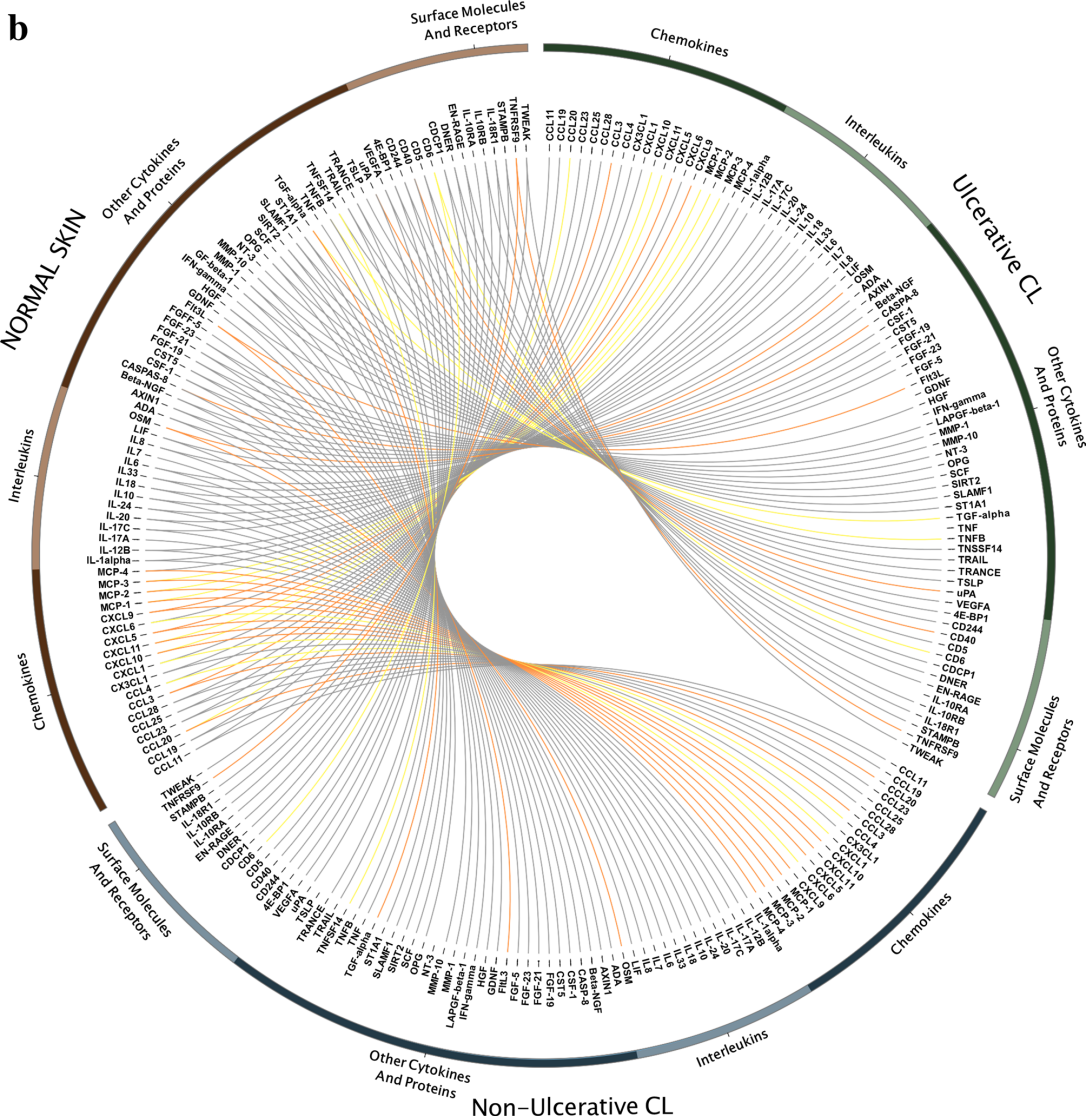
Inflammatory proteins expression in the lesions of the UCL and NUCL patients compared with healthy skins. The supernatants from UCL, NUCL and healthy biopsies cultured in RPMI, were analysed using Olink Inflammation panel. (**a**) NPX values for proteins with significant expression (*p* < 0.05) in the all lesions (full squares = UCL, open squares = NUCL) relative to healthy skin (Full circles). The inflammatory proteins were categorized into: CC and CXC chemokines family, Surface Molecules and Receptors and other cytokine and inflammatory proteins. (**b**) Circos plot displaying the inflammatory proteins in the UCL, NUCL and healthy samples. Results were analyzed with a *t* test/Mann–Whitney test, and Šidák^74^ adjusted *p*-values < 0.05 were considered statistically significant. The Circos plot in (**b**), generated using CIRCOS v 0.69-6^75^, visualizes the similarities and differences between UCL and NUCL patients. Red lines (*p* < 0.01), orange lines (*p* < 0.05) and yellow lines (*p* < 0.1).

The GSEA results revealed significant transcriptional changes of several pathways involved in inflammatory responses in the CL lesions, including “Chemokine receptors bind chemokines”, “Signaling by Interleukins”, “IL-10 signaling”, “IL-4 and IL-13 signaling” “IFN- γ signaling”, “IFN-α/β signaling”, and “IFN Signaling” (Fig. 4a,b). To this end, the expression of 92 proteins, involved in inflammatory responses, was assessed in the skin lesion biopsies of all patients (Fig. 5a), and is displayed separately for UCL and NUCL patients relative to healthy skin samples (Fig. 5b). The analysis showed that 18 and 19 inflammatory proteins were significantly up-regulated in the lesions from UCL and NUCL patients compared to those of healthy skin, respectively. Among these proteins, both patient groups had high expression of the inflammatory chemokines CCL3, CCL20, CXCL5, CXCL9, CXCL10, CXCL11, monocyte chemotactic protein (MCP) 1, MCP2, MCP3, Oncostatin-M (OSM), T cell surface glycoprotein CD6 isoform (CD6), transforming growth factor alpha (TGF-alpha) and tumor necrosis factor ligand superfamily member 9 (TNFSF9). Further, Caspase-8 (CASP8), tumor necrosis factor beta (TNFB), urokinase-type plasminogen activator (uPA) and CD40 were uniquely up-regulated in UCL patients. The expression of inflammatory chemokines (CCL4, CXCL1, CXCL6 and MCP-4) and TNF ligand superfamily member 14 (TNFSF14) were exclusively up-regulated in the NUCL lesions.

The dendritic cell development cytokine Flt3L and the immune-regulatory cytokine IL-33 were the only two proteins that showed down-regulation in the lesions of both UCL and NUCL patients compared to the healthy skin.

### *L. tropica* specific IgG antibody, ADCP and ADNP in sera and skin lesions of CL patients

**Figure 6:**
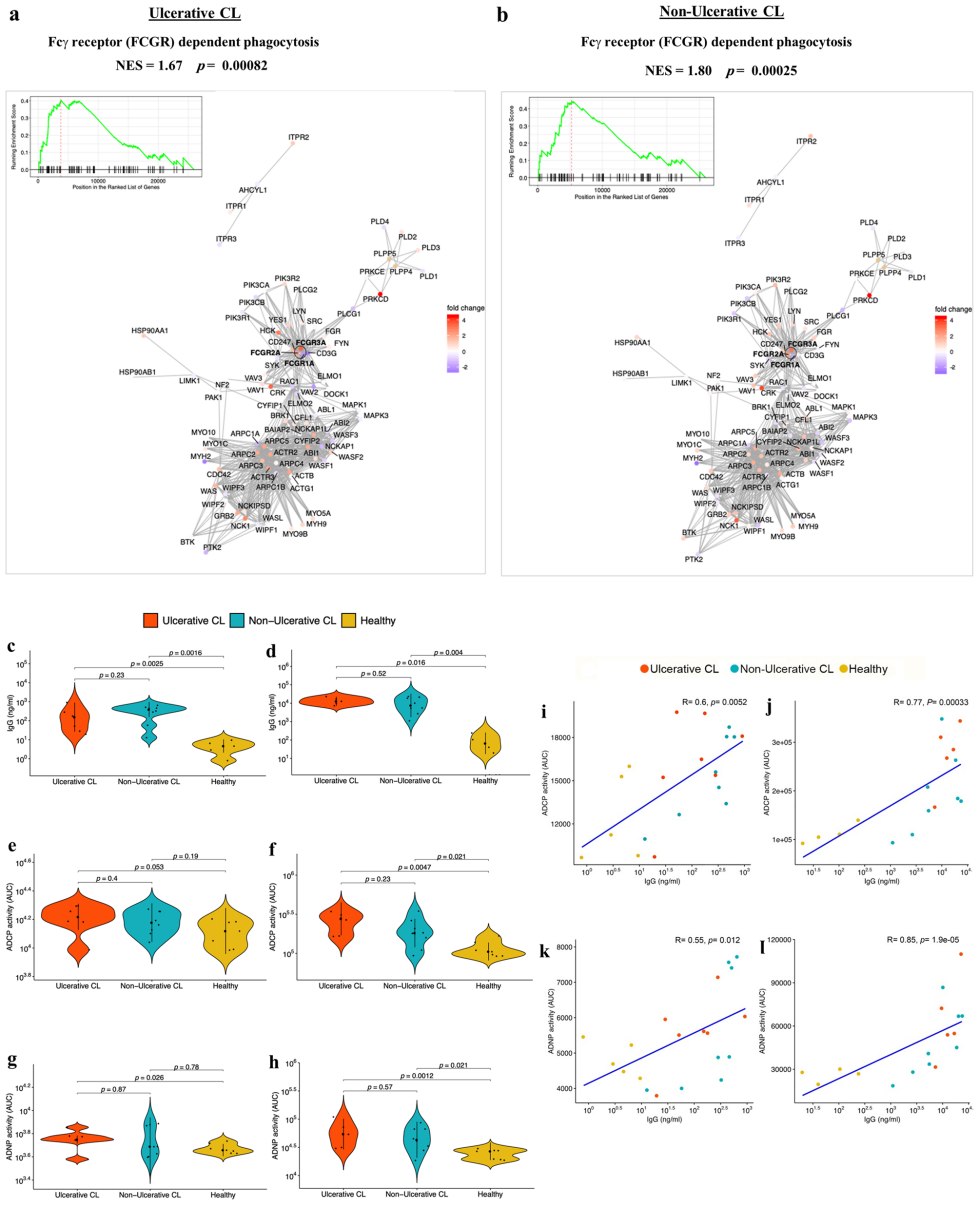
The enriched “FcγR dependent phagocytosis” gene pathway and antibody-dependent phagocytosis assays reveal antibody-dependent cellular phagocytosis (ADCP) and antibody-dependent neutrophil phagocytosis (ADNP) in the lesions of CL patients. Gene networks related to “FcγR dependent phagocytosis” pathway was enriched in the GSEA in skin lesions from UCL (**a**) and NUCL (**b**) patients. The dot colors indicate fold change in the gene expression as stated in the map. Violin plots show *L. tropica*-specific IgG antibody levels (ng/mL) in skin biopsy supernatants (**c**) and serum samples (**d**), ADCP activity in skin biopsy supernatants (**e**) and serum samples (**f**), as well as ADNP activity in skin biopsy supernatants (**g**) and serum samples (**h**). The phagocytic activity (AUC) was measured by the uptake of *L. tropica*-specific antibody-bead complexes by either human peripheral neutrophils or THP-1 cells, using flow cytometry. Spearman correlation between IgG antibody levels and ADCP, in skin biopsies (**i**) and serum samples (**j**), as well as between IgG antibody levels and ADNP, in skin biopsies (**k**) and serum samples (**l**). All figures were created using R version 3.5.1.

Our GSEA analysis revealed enrichment of pathways involved in FcγR dependent phagocytosis (Fig. 6a,b) along with the neutrophil degranulation pathway^28^ in both UCL and NUCL patients. Given that phagocytic cup formation genes were uniquely enriched in NUCL, we hypothesized that antibody-dependent phagocytosis may be elevated in NUCL. Guided by this, we evaluated *L. tropica* specific IgG antibody levels and antibody-dependent phagocytosis mediated by monocytes and neutrophils in the skin biopsy supernatants and serum samples obtained from the UCL and NUCL patients as well as those of healthy controls.

The UCL and NUCL patients had significantly higher levels of *L. tropica*-specific IgG antibodies, both in their lesion biopsy supernatants (UCL *p* = 0.0025, NUCL *p* = 0.0016) (Fig. 6c) and serum samples (UCL *p* = 0.016, NUCL *p* = 0.004) (Fig. 6d), compared with those of the healthy individuals. *L. tropica*-specific antibody-dependent phagocytosis assays, including antibody-dependent neutrophil phagocytosis (ADNP) and antibody-dependent monocyte phagocytosis (ADCP) were carried out using primary human neutrophils and the monocytic THP-1 cells, respectively. The lesion biopsy supernatants from UCL displayed significantly higher ADNP activity (*p* = 0.026) than that of the healthy individuals (Fig. 6g). However, ADCP activity in UCL and NUCL in the lesion supernatants did not significantly differ from the control group (Fig. 6e). Serum samples from the UCL and NUCL patients showed significantly stronger ADCP and ADNP activities compared with those of the healthy control group (UCL-ADCP *p* = 0.0047, NUCL-ADCP *p* = 0.021, UCL-ADNP *p* = 0.0012, NUCL-ADNP *p* = 0.021) (Fig. 6f,h).

A statistically significant correlation was observed between ADCP (R = 0.83, *p* = 0.015) and ADNP (R = 0.9, *p* = 0.0046) activities and *L. tropica*-specific IgG antibodies in the lesion biopsies of the NUCL patients. Only ADNP activity was found to correlate with *L. tropica*-specific IgG antibodies in the lesion biopsies of the UCL patients (R = 0.71, *p* = 0.088). The ADNP activity also showed statistical significance with *L. tropica*-specific IgG antibodies in the sera of the NUCL patients (R = 0.83, *p* = 0.015). However, statistically significant correlations were observed between *L. tropica*-specific IgG antibodies and ADCP/ADNP activity in the lesion biopsies (ADCP: R = 0.6, *p* = 0.0052, ADNP: R = 0.55, *p* = 0.012) and sera (ADCP: R = 0.77, *p* = 0.00033, ADNP: R = 0.85, *p* = 1.9e−05) of CL patients when results from both NUCL and UCL groups were considered (Fig. 6i–l).

Next, parasite burden in the UCL and NUCL lesion biopsies was quantified using qPCR, and the results were correlated with *L. tropica*-specific IgG antibodies level as well as ADCP and ADNP activities. An inverse correlation between the parasite load and specific IgG antibodies (biopsy: R = − 0.6, *p* = 0.12; serum: R = − 0.64, *p* = 0.086), ADCP (biopsy: R = − 0.5, *p* = 0.21; serum: R = − 0.48, *p* = 0.23) and ADNP (biopsy: R = − 0.71; *p* = 0.047, serum: R = − 0.62, *p* = 0.1) was observed in the lesion biopsy and serum samples derived from the NUCL patients. These parameters did not show statistically significant correlations in the UCL group (IgG/biopsy: R = 0.12, *p* = 0.78; IgG/serum: R = -0.3, *p* = 0.62; ADCP/biopsy: R = 0.14, *p* = 0.74; ADCP/serum: R = 0.14, *p* = 0.79; ADNP/biopsy: R = 0.29, *p* = 0.49; ADNP/serum: R = 0.14, *p* = 0.79). Although when all UCL and NUCL patients were considered as a single group, an inverse correlation was observed between the parasite load and *L. tropica*-specific IgG antibody levels (biopsy R = − 0.35, *p* = 0.18; serum R = − 0.48, *p* = 0.097), ADCP (biopsy R = − 0.15, *p* = 0.57; serum R = − 0.18, *p* = 0.54) and ADNP (biopsy R = − 0.29, *p* = 0.27; serum R = − 0.28, *p* = 0.33) in the lesion biopsy (Fig. S3a–c) and serum samples (Fig. S3d–f).

## Discussion

We herein applied whole genome RNA sequencing along with high-throughput PEA assay and antibody-dependent phagocytosis assays combined with systems biology approaches to pinpoint molecular signatures of human response to *L. tropica* infection in the lesions of UCL and NUCL patients.

Using GSEA analysis, we identified unique and common enriched pathways in the lesions of the UCL and NUCL patients. The results showed that the majority of the NUCL pathways were shared between NUCL and the UCL group. Several of the common enriched pathways were involved in the host immune response, including “TLR pathways”, “IFNs signaling”, “TCR complex”, “FcγR-dependent phagocytosis” and “Notch signaling”. Depending on the immune status of the host and the *Leishmania* species, TLRs may have protective or deleterious effect on disease outcome^29,30^. IFNs can have beneficial or deleterious effect on the elimination of the parasite by macrophages, depending on the type of IFN produced and the stage of infection^31,32^. The activation of macrophage leading to killing *Leishmania* is initiated by ROS production and activation of Th1 immune responses^33^. The enriched pathways related to T cells were mostly related to T-cell activation, with exception for PD-1 signaling that serves as a negative regulator of T cells, and mediates T cell exhaustion^34,35^. Co-stimulation by CD40, as a member of TNFR superfamily, contributes to diverse immune responses against different species of *Leishmania*^35^. Ligand-independent Notch activation contributes to optimal T-cell proliferation and activation^36,37^, and as such is likely to contribute to immunity to CL. It is noteworthy that a previous study showed that signaling pathways related to TLR and T cell receptor in human macrophages infected with *L. major* were up-regulated, whereas Fc gamma R-mediated phagocytosis was down-regulated in KEGG enriched pathways^38^.

The observed similarities in the molecular signatures of UCL and NUCL could, at least in part, be explained by the fact that the biopsies were collected during the acute stage of the disease when common immune mechanisms (mostly of inflammatory nature) are in play. Nevertheless, our analyses also identified unique molecular pathways and inflammatory proteins in UCL and NUCL.

In the UCL group, 55 pathways were uniquely enriched and most of these pathways were categorized into “gene expression” and “signal transduction” pathways (Fig. S2a). It is known that epigenetic regulation of gene expression and DNA methylation play important roles in the function of macrophages, which in turn may impact the survival of *Leishmania* in macrophages^26,27^. Several enriched signal transduction pathways, including “signaling by nuclear receptors”, “WNT”, “GPCR”, “Notch pathways” and “RHO GTPase effectors” were shown to impact the cytoskeletal and membrane architecture of the host cells during parasite infection^39–42^. There is currently a dearth of knowledge regarding transcription and post-transcription modifications of host genes induced by the *Leishmania* species^26^.

Further, GSEA pathways involved in “induction of B-cell receptors” and “NF-κB activation in B cells” involved in B-cell development and activation^43^ were positively enriched in the UCL group. Previous human studies have suggested that B-cell activation can lead to the exacerbation of CL disease^19,44^. The involvement of B-cell responses to *Leishmania* appears to be complex, and whether they participate in parasite persistence or killing remains unclear. Only four unique biological pathways were positively enriched in the NUCL group, including “Initiation of Nuclear Envelope Reformation”, “Nuclear Envelope Reassembly” during mitotic cell cycle”^45^, “apoptosis induction by P53”^46^ “and actin dynamic during phagocytosis”^47^.

Our GSEA analysis also showed enriched pathways for FcγR dependent phagocytosis in both UCL and NUCL lesions. Antibody-dependent cellular phagocytosis and antibody-dependent cellular cytotoxicity are two main mechanisms involved in FCγR-mediated pathogen clearance^48^. “Neutrophil degranulation pathway” that may be associated with antibody production^28^ was also positively enriched in the lesions of both UCL and NUCL patients. Neutrophils are the first inflammatory cells involved in pathogen recognition during early stage of infection. These cells can modulate the immune response against *Leishmania* and can contribute to the clearance of parasite by various mechanisms^49^. Carlsen et al. showed the potential function of neutrophils for limiting *L. braziliensis* growth via direct internalization of the parasite or enhancing macrophage activity^50^. The presence of a significant ADNP activity in the lesions and sera of the UCL patients in the lesions suggests that ADNP may play a role in parasite killing. However, further studies are needed to determine the role of ADNP in host response to CL.

In line with the GSEA data, significant levels of chemokines involved in inflammation and recruitment of immune effector cells, including CCL3, CCL20, CXCL5, CXCL9, CXCL10, CXCL11, MCP1, MCP2, and MCP3 were commonly observed in the skin lesions of both UCL and NUCL patients. This inflammatory milieu contributes to the influx of innate immune cells, including monocytes, neutrophils and antigen presenting cells to skin, which may in turn facilitate parasite phagocytosis by neutrophils and monocytes, and mediates inflammation-induced skin pathology^51,52^. Further, OSM protein had the highest ratio of expression in skin lesions patients compared to the healthy skins. OSM is a pleiotropic cytokine that belongs to the IL-6 family, and is mainly secreted by T cells, monocytes, macrophages, dendritic cells (DCs) and neutrophils. OSM has been shown to play an important role in regulation of keratinocyte differentiation and down-regulation of expression of epidermal genes in human skin inflammatory disorders, such as atopic dermatitis and psoriasis^53–55^. Further, our results showed negative enrichment of several genes associated with keratinocytes and the cornified envelope (the outer layer of the epidermis) such as Keratins, filaggrin, loricrin, desmogleins, desmocollins, small proline rich proteins, late cornified envelope and the epidermal protease kallikreins in the lesions of both UCL and the NUCL patients (Fig. S2e–h). These results warrant further exploration of the role of OSM in the epidermal differentiation and wound healing in CL patients.

In line with our previous findings^22^, striated muscle contraction pathway and related genes, such as actin and myosin, were found to be down-regulated in both lesion types (Fig. S2i,j). It has previously been shown that F-actin and myosin play an important role in host cytoskeleton modification required for the interaction between macrophages and *L. braziliensis*^56^.

Few proteins were uniquely expressed in the UCL group, among which CASP8 plays a role in macrophage apoptosis^57^. This may explain, at least in part, the weak ADCP activity observed in the skin lesions of the UCL patients compared to that of the NUCL group. uPA protein is involved in the extracellular matrix degradation^58^, which may contribute to the extensive tissue damage observed in the UCL patients. In *L. braziliensis* patients, the positive correlation was observed between TNF level and ulcer size and suggested TNF inhibitors can improve healing process in CL patients with severe lesions^59^.

In the biopsies of the NUCL patients, five unique inflammatory proteins were up-regulated, including the monocyte chemo-attractants CCL4 and CCL13 (MCP-4) and the neutrophil chemo-attractants CXCL1 and CXCL6, as well as TNFSF14. The influx of neutrophils and monocytes to the skin in response to the chemokine gradient could facilitate their antibody-mediated phagocytosis function. TNFSF14 (a member of the TNF superfamily) may have contradicting impacts on the parasite growth i.e., activation of anti-parasitic immune mechanisms, via DC induction of IL-12 production, or increased parasite growth by reduction of IL-12, all depending on engagement with its receptors and the down-stream signaling^60,61^.

Our data also showed that the parasite load was lower in the UCL lesions, albeit not statistically significant, than in NUCL. In line with this finding, Tasew et al.^62^ found that tissue destruction in localized CL was associated with a low parasite load in an inflammatory environment with a strong Th1 responses, whereas diffuse CL was defined by a high parasite load and an inefficient T-cell responses.

In summary, we herein employed RNA sequencing, a multiplex inflammatory protein assay, ADCP/ADNP assays and analysis of the parasite burden in order to discriminate between UCL and NUCL in anthroponotic *L. tropica* infection. These results can enhance our knowledge of human responses to anthroponotic CL beyond the state-of-the-art, and inform rational design of novel interventions strategies to counter CL in humans.

## Methods

### Patient population and diagnosis

The patients had been referred to the CL special clinic at Mashhad Medical School in Mashhad, a highly endemic area in Iran for *L. tropica*. The CL lesions were classified according to their clinical presentation as ulcerated lesion (UCL) with eruptive overlying epidermis covered with a crust or non-ulcerated lesion (NUCL) with intact epidermis^63^. All CL patients had an active lesion for a maximum of 1 year, and had no prior anti-leishmanial treatment. A total of eight UCL and nine NUCL patients were enrolled in the study. Healthy skin samples were taken from eight volunteers undergoing esthetical surgery at the Noor Eye hospital in Tehran, Iran with no history of leishmaniasis or other skin disorders.

The criteria for patient enrolment include clinically verified characteristics of CL skin lesion and a positive result from the diagnostic *L. tropica* specific PCR using samples obtained from a non-invasive, tape disc- based sampling method. Briefly, the first PCR targeting the kDNA minicircle of *Leishmania* was performed, followed by a second PCR for *Leishmania* species identification as described by Taslimi et al^23^.

### Skin lesion biopsies and serum samples

From each CL patient and healthy individual, two skin biopsies and 2 mL whole blood were collected. In the case of patients, the biopsies were collected at the border of the lesion using a 2 mm punch. First, punch biopsies were cut in half and immediately placed in RNA later (Qiagen GmbH, Hilden, Germany) and then stored at − 20 °C. One half was used for RNA sequencing and the other half for parasite quantification.

The second punch biopsy was cultured for 48 h at 37 °C with 5% CO_2_ in RPMI supplemented with 10% human AB serum (Sigma), 2 mM glutamine, 10 mg/mL gentamycin, 4 mM Hepes, 23 mM bicarbonate and 0.1% 2ME. The culture supernatants were collected and stored at − 80 °C.

Blood samples were incubated at 37 °C for 1 h followed by centrifugation for 10 min at 3000 rpm, and the retrieved serum samples were stored at − 80 °C. The workflow of sampling procedures and the downstream assays are depicted in Fig. 1.

### Preparation of *L. tropica* lysate

*L. tropica* (MOHM/IR/09/Khamesipour-Mashhad) strain was cultured in Medium 199 (M199; Sigma) supplemented with 10% heat-inactivated fetal calf serum (FCS, Gibco), 40 mM HEPES (Sigma), 5 mg/mL hemin, 0.1 mM adenosine (Sigma) and 50 µL/mL gentamicin (Sigma), and incubated at 26 °C. Parasites were collected by centrifugation (10 min at 3000 rpm) and *L. tropica* lysate was prepared using 3 × 10^8^ parasite/mL with 10 times rapid freeze–thaw cycles in liquid nitrogen and 37 °C water bath, followed by storage at − 80 °C.

### Primary human neutrophils and monocytes cell line

Human neutrophil cells were obtained from the white blood cells of healthy human donor peripheral blood followed by lysing the red blood cells with Ammonium–Chloride–Potassium lysis buffer. White blood cells were washed with PBS and cultured in RPMI 1640 supplemented with 10% fetal bovine serum, l-Glutamine, and Pen/Strep. THP-1 cells (ATCC^®^ TIB-202™) were cultured in RPMI 1640 supplemented with 10% fetal bovine serum, l-Glutamine, Pen/Strep, and 55 μM beta mercaptoethanol.

### Parasite quantification assay in skin lesions from CL patients

The skin biopsies in RNAlater, were minced with a sterile scalpel and then incubated at 56 °C for 3 h in PBS buffer containing proteinase K (Qiagen, Germany). DNA extraction was performed using a QIAamp DNA mini kit for lysed tissues (Qiagen, Germany), according to the manufacturer’s instructions. The concentration and purity of isolated DNA samples were assessed using a Nanodrop spectrophotometer (ND-1000, USA).

Quantitative real-time PCR (qPCR) was used to measure the parasite burden. Briefly, for each PCR reaction, 20 ng DNA template, 1 µM SYBR Green PCR master mix (Qiagen, Germany), 10 pmol of kDNA1-F (5′-GGGTAGGGGCGTTCTGC-3′) and kDNA1-R (5′-TACACCAACCCCCAGTTTG-3′) primers, and Rox dye (1:200 dilution) were used in a total volume of 20 µL. Cycling conditions included 5 min at 95 °C, followed by 40 cycles of 30 s at 95 °C, 2 min at 60 °C and finally 1 min at 72 °C.

TATA-binding protein (TBP) gene was used as normalizer with similar reaction conditions. The primers for TBP were forward (5′-AGTTGTCATACCGTGCTGCTA-3′) and reverse (5′-TTCTCCCTCAAACCAACTTGTCA-3′). Amplification was performed in an Applied Biosystems 7500 machine (USA). Delta Ct (ΔCT) values were determined using Ct_kDNA_ − Ct_TBP_, and the fold change calculated according to the 2^-ΔΔCT^ formula^24^. For each sample, PCR was performed in duplicate. Mann–Whitney test was applied using GraphPad Prism software (version 6.0; GraphPad Software, San Diego, CA, USA) to determine the statistical significance of the observed differences between UCL and NUCL groups. The mean and the standard error of the mean were calculated for each group.

### RNA isolation and cDNA library preparation

Skin biopsies in RNAlater were homogenized in QIAzol Lysis buffer using stainless steel beads and TissueLyser II (Qiagen, GmbH, Hilden, Germany). Total RNA was isolated using miRNeasy Mini Kit (Qiagen, GmbH, Hilden, Germany), according to the manufacturer’s protocol. RNA concentrations were measured using Nanodrop spectrophotometer (Thermo Scientific, USA), followed by RNA integrity (RIN) evaluation by Agilent 2200 TapeStation (Agilent Technologies, USA). RNA samples with a 260/280 ratio approximating 2 and RIN > 7 were used in RNA sequencing analyses. One patient sample and one control sample were excluded from subsequent analyses due to low RIN values.

Ribosomal RNA depletion and cDNA library preparation were performed using the IlluminaTruSeq Stranded Total RNA Sample Preparation Kit with Ribo-Zero Gold (San Diego, California, USA). Final libraries were evaluated using Agilent 2200 Tape Station and Qubit^®^Fluorometer (Thermo Fisher-Scientific).

### RNA sequencing and data analysis

Final pool of libraries was run on a NextSeq 500 platform (2 × 75, high output, 1.75 pM), generating a minimum of 30 M reads per sample. Sequence quality was assessed using FastQC (v 0.11.2) followed by merging the reports using multiQC (v 0.9). Prinseq (v 0.20.3) was used to remove any remaining Illumina adapter sequences from reads and to trim bases off the start or the end of a read when the quality score fell below a threshold of 20. Star (v 2.5.2b)^64^ was applied to align reads to the human genome (v hg19/GRCh38) obtained from the UCSC genome browser (https://genome.ucsc.edu). The abundance of reads mapping to each gene feature in the aligned genome was determined using HTSeq (v 0.6.1p1)^65^. Subsequent analyses were performed in R (Version 3.5.1, https://www.r-project.org/)^66^. A list of differentially expressed genes (DEGs) between the patients and healthy group was generated using the R package DESeq2 (v 1.22.1)^67^. Genes were considered significantly differentially expressed if False Discovery Rate (FDR) < 0.05. Heat maps were created using *pheatmap* (version 1.0.10).

### Functional analysis of genes and gene clusters

The R packages clusterProfiler (v 3.8)^68^ and ReactomePA^69^ were applied to run Gene Set Enrichment Analysis (GSEA)^70^, and DOSE (v3.8.2). R package^71^ was used to visualize functional profiles of genes and gene clusters. All genes were subjected to the GSEA analysis, while curated gene sets of Reactome pathways were used to identify significantly enriched pathways based on a FDR < 0.01, and enrichment score (ES) is reported.

### Multiplex PEA in the supernatant of skin lesions from CL patients and healthy skin biopsies

The supernatants, derived from biopsies cultured in RPMI, were analysed using Olink Inflammation panel (Olink Proteomics, Uppsala, Sweden). The expression levels of the 92 cytokines and chemokines present in this Olink panel, were measured based on PEA technology^72^. In short, paired oligonucleotide-labelled antibodies were used to detect target proteins in the 1-µL supernatant samples. Upon recognition of a target antigen protein, by a pair of labels, the DNA oligonucleotides on the antibodies will be amplified through PEA enzyme and PCR reagents, and quantified using a microfluidic PCR platform. The biomarkers are presented in normalized protein expression (NPX) arbitrary units on a Log_2_ scale. The Sidak T test method was used to calculate the *p*-values in UCL and NUCL lesion types and *p*-values less than 0.05 were considered significant.

### Antibody-dependent neutrophil phagocytosis (ADNP)

*L. tropica* lysate was biotinylated and coupled to yellow-green Neutravidin beads (Life Technologies). Samples were diluted five-fold (1:100–1:2500 for serum samples and 1:10–1:500 for biopsy supernatants) in culture medium and incubated with *L. tropica* lysate-coated beads for 2 h at 37 °C. Freshly isolated white blood cells from healthy human donor peripheral blood (5 × 10^4^ cells/well) were added and incubated for 1 h at 37 °C. Cells were stained for expression of CD66b (Clone G10F5; Biolegend, San Diego, CA), CD3 (Clone UCHT1; BD Biosciences), and CD14 (Clone MφP9; BD Biosciences), fixed with 4% paraformaldehyde, and analyzed by flow cytometry. Neutrophils were defined as SSC-A^high^ CD66b^+^, CD3^-^, CD14^−^. A phagocytic score was determined using the following formula: (percentage of FITC^+^ cells) × (geometric mean fluorescent intensity, gMFI, of the FITC^+^ cells)/10,000^73^.

### Antibody-dependent cellular phagocytosis by human monocytes (ADCP)

*L. tropica* lysate-coated beads were generated as described above for ADNP. Samples were diluted five-fold in culture medium (1:200–1:5000 for serum samples; and 1:25–1:625 for biopsy supernatants) and incubated with *L. tropica* lysate-coated beads for 2 h at 37 °C. Unbound antibodies were removed by centrifugation prior to the addition of THP-1 cells at 2.5 × 10^4^ cells/well for 18 h at 37 °C. Cells were fixed with 4% paraformaldehyde and analyzed by flow cytometry. A phagocytic score was determined as described above.

### *L. tropica*-specific IgG antibodies assessment in skin biopsy supernatants and sera

MaxiSorp microplates (Sarstedt) were coated with *L. tropica* lysate overnight. The plates were blocked with 1% IgG-free bovine serum albumin (Sigma Aldrich) in PBS for 1 h to reduce non-specific antigen–antibody interaction. The coated plates were incubated for 1 h with biopsy supernatants (diluted 1:4) or serum samples (diluted 1:125), after which goat-anti-human IgG Fc antibodies (Sigma), conjugated with horseradish peroxidase, were added and incubated in dark for 1 h. The plates were developed with *o*-phenylenediamine dihydrochloride and absorbance read at 490 nm (BioTek, ELx800).

### Ethics statement

This study was approved by the Ethics Committee of the Pasteur Institute of Iran (IR.PII.REC.1398.044), and all methods were performed in accordance with the relevant guidelines and regulations. All CL patients and healthy individuals were given information about the project and written informed consent was obtained from all study participants for the collection of samples and subsequent analysis.

## Supplementary information

Supplementary Information 2.

Supplementary Information 2.

Supplementary Information 3.
