# Supplementary material for: Molecular signatures of anthroponotic cutaneous leishmaniasis in the lesions of patients infected with Leishmania tropica
Source: Sci Rep. 2020 Oct 1;10:16198. doi: 10.1038/s41598-020-72671-7 (PMC7529897; doi:10.1038/s41598-020-72671-7)
Supplement: Supplementary file 1 — Supplementary Information 2. [file 41598_2020_72671_MOESM1_ESM.docx]

**Molecular signatures of anthroponotic cutaneous leishmaniasis in the lesions of patients infected with *Leishmania tropica***

Nasrin Masoudzadeh^1^, Malin Östensson^2^, Josefine Persson^2^, Vahid Mashayekhi Goyonlo^3^, Christopher Agbajogu^2^, Yasaman Taslimi^1^, Reza Erfanian Salim^4^, Farnaz Zahedifard^1^, Amir Mizbani^5^, Housein Malekafzali Ardekani^1^, Bronwyn M. Gunn^6^, Sima Rafati^1^*****, Ali M. Harandi^2,7^*****

^1^Department of Immunotherapy and *Leishmania* Vaccine Research, Pasteur Institute of Iran, Tehran, Iran.

^2^Department of Microbiology and Immunology, Institute of Biomedicine, Sahlgrenska Academy, University of Gothenburg, Gothenburg, Sweden.

^3^Cutaneous Leishmaniasis Research Center, Mashhad University of Medical Sciences, Mashhad, Iran

^4^Noor Eye hospital, Tehran, Iran.

^5^ETH Zurich, Switzerland.

^6^Ragon Institute of MGH, MIT, and Harvard University, Cambridge, MA 02139, USA. Present address: Paul G. Allen School of Global Animal Health, Washington State University, Pullman WA 99164, USA.

^7^Vaccine Evaluation Center, BC Children’s Hospital Research Institute, The University of British Columbia, Vancouver, Canada

***Corresponding authors: these authors contributed equally to the work**

Sima Rafati: s_rafati@yahoo.com; sima-rafatisy@pasteur.ac.ir

Ali M. Harandi: ali.harandi@microbio.gu.se


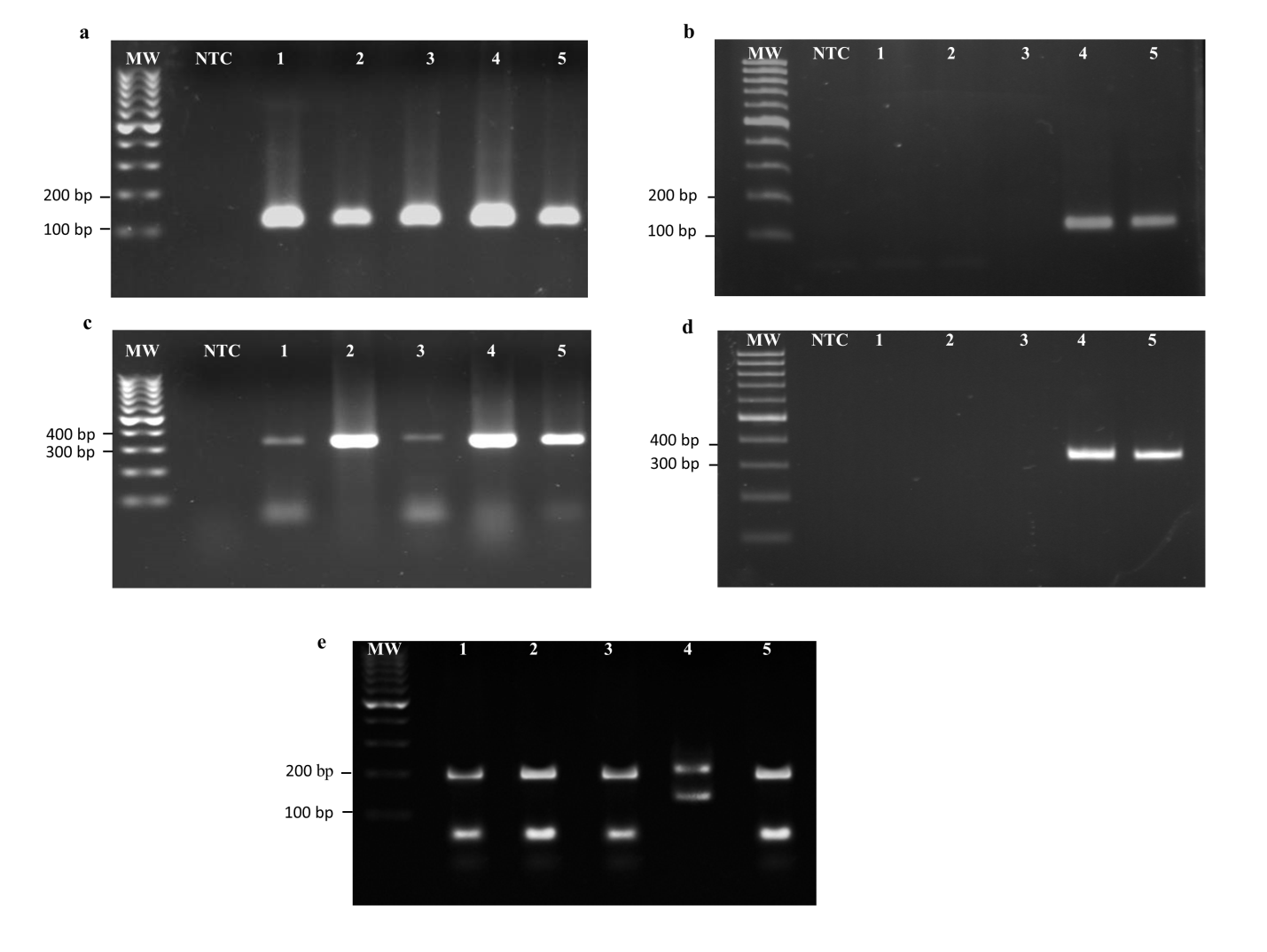


Fig S1


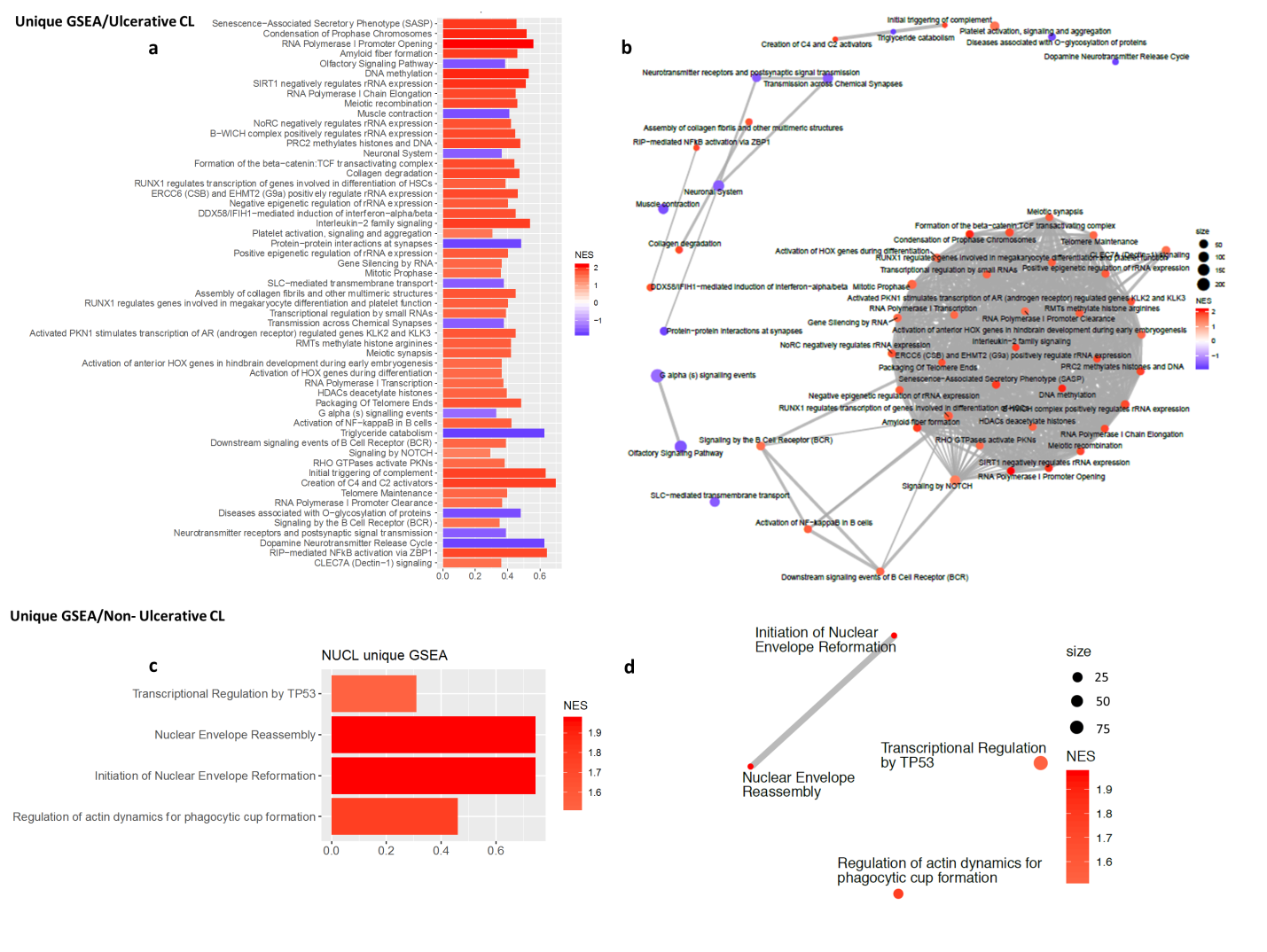


Fig S2a-d


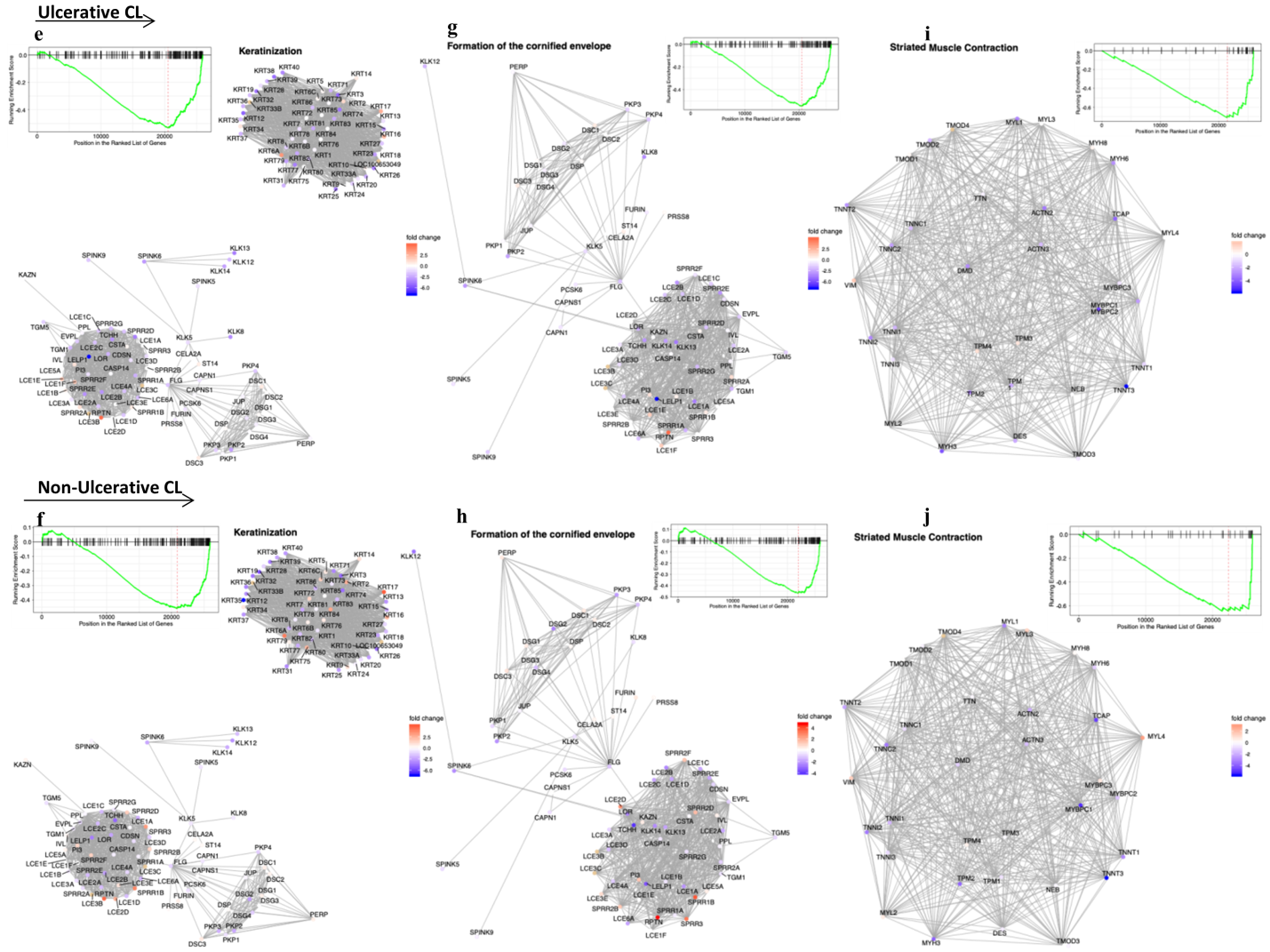


Fig S2e-j


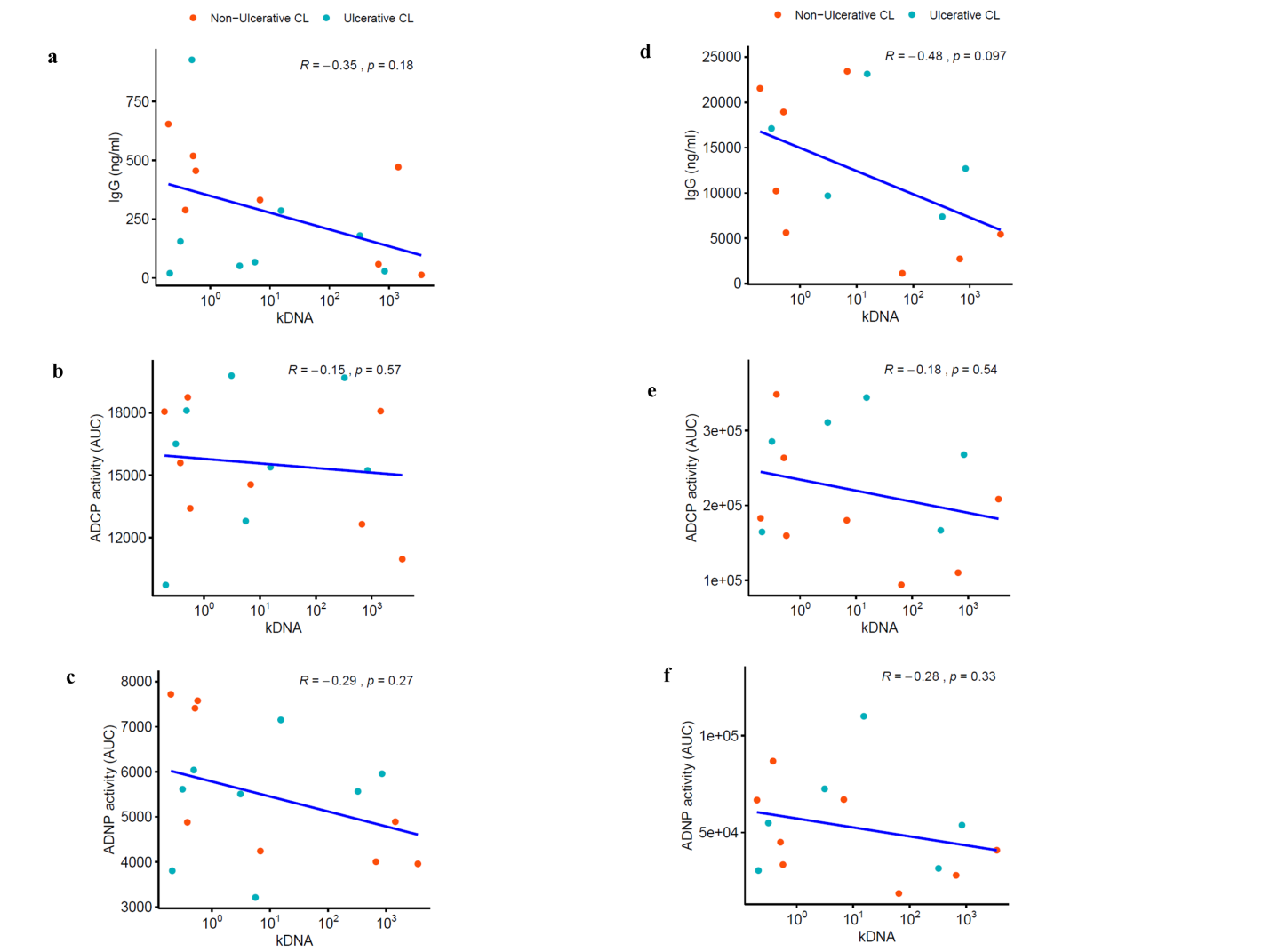


Fig S3

| Characteristic | UCL  N = 8 | NUCL  N = 9 |
| --- | --- | --- |
| Gender |  |  |
| Female | 4 (50 %) | 6 (66.6 %) |
| Male | 4 (50 %) | 3 (33.3 %) |
| *Age | 43.62 ± 3.90 | 48.44 ± 6.86 |
| *Lesion size (cm^2^) | 26.33 ± 9.95 | 15.66 ± 7.31 |
| *illness duration (month) | 4.25 ± 1.20 | 4.16 ± 1.01 |
| Lesion number  1  ≥2 | 4 (50%)  4 (50%) | 4 (40%)  5 (60%) |
| *Plus-minus values are means ± SE | |  |

Table S1. Patient characteristics
