# Supplementary material for: Molecular signatures of anthroponotic cutaneous leishmaniasis in the lesions of patients infected with Leishmania tropica
Source: Sci Rep. 2020 Oct 1;10:16198. doi: 10.1038/s41598-020-72671-7 (PMC7529897; doi:10.1038/s41598-020-72671-7)
Supplement: Supplementary file 3 — Supplementary Information 3. [file 41598_2020_72671_MOESM3_ESM.docx]

**Supplementary Information**

**Fig S1. Differential diagnosis of cutaneous leishmaniasis patients using a kDNA-based method.**

Diagnosis was performed using the tape disc, non-invasive sampling technique, and two diagnostic PCRs. The first PCR was targeting the kDNA1 of Leishmania species and the second PCR was for species identification. The PCR products were subjected to RFLP analysis by digesting the ITS1 PCR product with *HaeIII* enzyme.

kDNA (a, b) and ITS1 amplification (c, d) in the CL patients (lanes 1-3) (a, c) and healthy individuals (lanes 1-3) (b, d). Standards for *L*. *tropica* (lane 4) and *L. major* (Lane 5) were included in all gels. (e) Restriction fragment length polymorphism technique was utilized, using digestion enzyme *HaeIII*, for the ITS1 PCR product. A 100-bp ladder was included on all gels as a molecular weight (MW) size marker.

**Fig S2. Unique enriched GSEA pathways and common down-regulated enriched GSEA pathways in UCL and NUCL lesions.**

(a) Bar plot and (b) enrichment map plot of GSEA displaying the unique pathways of *L. tropica*-infected patients in the UCL group. Bar plot (c) and enrichment map plot (d) of GSEA displaying the unique pathways of *L. tropica*-infected patients in the NUCL group. The color and length of the boxes represent normalized enrichment scores (NES) and the number of genes mapped to the indicated pathways, respectively. Significant down-regulated pathways (adjusted *p*-value <0.05), shared between UCL (e, g, i) and NUCL (f, h, j), include Keratinization (e, f), Formation of the cornified envelope (g, h), and Stratified muscle contraction (i, j). The fold change of the significant genes associated with these pathways, are represented as stated in the color map. For the pathway analysis ReactomePA R package version 1.28.0 was used[^69^](#_ENREF_69). This package version uses Reactome version 70 (<https://reactome.org/about/news/142-version-70-released>). All figures were created using R version 3.5.1.

**Fig S3. Correlation analysis between parasite burden, *L. tropica* specific antibody levels and antibody dependent phagocytosis.**

Spearman correlation analysis between Log_10_ of qPCR-verified levels of parasite load (kDNA) and (a, d) *L. tropica* specific IgG antibody levels, (b, e) ADCP, and (c, f) ADNP. Results from skin biopsy samples (a-c) and serum samples (d-f) are presented. These plots were created using R package ggpubr 0.4.0.

**Table S1.** **Patient characteristics and clinical data.**

Clinical profile of the UCL and NUCL patients, including sex, age, lesion size, illness duration and number of lesions, are presented.

**Table S2. All GSEA pathways enriched in UCL and NUCL lesion types.**

GSEA was performed on the gene expression data from the UCL and NUCL lesions compared with the healthy skins to pinpoint biological functions related to groups of genes by a Reactome database. GSEA statistic parameters, including ES, NES and adjusted *p*-values, are presented for each pathway. The analysis identified 100 and 49 enriched Reactome pathways (adjusted *p*-values<0.05) in the skin lesions of UCL (first excel sheet) and the NUCL (second excel sheet) groups, respectively, when compared with healthy skin samples.
